# Supplementary material for: Are carbon emissions trading and green financial instruments synergistic? -Comprehensive quantitative research based on content analysis
Source: PLoS One. 2024 Mar 7;19(3):e0298601. doi: 10.1371/journal.pone.0298601 (PMC10919601; doi:10.1371/journal.pone.0298601)
Supplement: S1 Data — (ZIP) [file pone.0298601.s001.zip › Data/地级市Z_F工作报告.zip!/╔┬╬≈/╔╠┬σ╩╨/└┤╫╘╣1⁄2╓┌║┼í╛┬φ┐╦╩2╛▌═°í┐_2011.docx]

各位代表：

现在，我代表市人民政府向大会作工作报告，请各位代表连同《商洛市国民经济和社会发展第十二个五年规划纲要（草案）》一并审议，并请市政协各位委员和其他列席人员提出意见。

一、去年及“十一五”工作回顾

2010年是极不平凡的一年，全市上下以邓小平理论和“三个代表”重要思想为指导，深入贯彻落实科学发展观，克服国际金融危机和特大洪灾的不利影响，务实创新，埋头苦干，完成了市二届人大五次会议确定的目标任务。全市实现生产总值285.9亿元、增长14.9%，完成财政总收入21.24亿元、增长29.2%，地方财政收入12.01亿元、增长34.1%，城镇居民人均可支配收入和农民人均纯收入分别达到14811元和3605元，增长15.2%和20.1%，全社会固定资产投资290.07亿元、增长31.6%，社会消费品零售总额79.19亿元、增长18.6%。就业、社会保障、节能减排等主要指标全面完成，实现了“十一五”的完美收官。

回顾“十一五”，我们深深地感到，这五年是我市发展机遇最多、经济增长最快、发展质量最好、城乡面貌变化最大、群众得到实惠最多的五年，“十一五”规划确定的预期目标基本提前两年或一年实现，商洛的发展已经站在了一个新的历史起点上。

国民经济快速增长，综合实力显著提升。“十一五”期间，全市生产总值年均增长13.7%，去年经济总量是2005年的2.5倍。财政总收入年均增长32.8%，去年财政总收入是2005年的4.1倍，在全省的比重由0.97%提高到1.18%；地方财政收入年均增长37.3%，地方财政收入是2005年的4.9倍，在全省的比重由0.89%提高到1.25%；财政支出90.19亿元，是2005年的5.87倍，在全省的比重由2.4%提高到4.1%。去年城镇居民人均可支配收入是2005年的2.1倍，从占全省平均水平的85.1%提高到94.4%；农民人均纯收入是2005年的2.4倍，从占全省平均水平的73.7%提高到87.8%。金融机构各项存贷款余额分别较2005年增加247亿元、88亿元，增长 189.7％和135.1％。

发展方式有效转变，特色经济日益明晰。农业产业化经营水平不断提高，核桃、板栗、药材、茶叶、烟叶等特色产业快速发展，收入已占农业产值的63%，较2005年提高17个百分点。粮食连续四年增产，去年总产72.2万吨，增长6.8%。新型工业化进程加快，去年规模以上工业总产值达到168.78亿元，是2005年的5.1倍，年均增长38.6%；规模以上工业增加值是2005年的4.9倍，年均增长37.2%。循环经济成为破解环境约束、促进工业转型的主要途径，商丹园区被确定为省级循环经济园区，7个县域工业集中区被列为省级重点，累计入园企业111户；现代材料、生物医药和绿色食品产业支撑作用不断增强，太阳能光伏等战略性新兴产业快速发展，全市产值超亿元的企业发展到21户，较2005年增加19户。第三产业全面提速，服务业增加值年均增长14.8%；成功举办了中国首届秦岭生态旅游节，并被评为2010年全国最具潜力旅游节庆品牌，全市建成国家4A景区1个、3A景区2个，累计接待游客2722万人次，实现收入85.3亿元，较“十五”分别增长8倍和16倍。

项目建设加速推进，发展基础更加坚实。五年实施市级重大项目395个，完成投资568亿元，其中产业类项目143个、投资157亿元。累计完成全社会固定资产投资908.22亿元，是“十五”的6.2倍。交通条件根本改善，西柞、蓝商、商界、柞小、商漫高速公路建成通车，西商二线和洛商高速开工建设，全市高速公路通车里程351公里，商洛已进入西安1小时经济圈。地方交通投资231.64亿元，是“十五”的15倍，建成通乡公路1650公里、通达工程2320.4公里、通村水泥路6408公里，路网密度达66公里/每百平方公里。330千伏输变电鹿城新建、张村扩容等工程建成投运，农村电网改造持续推进，“户户通电”工程全面完成。“村村通电话”目标如期实现。西安至商洛天然气管道实现通气点火。农村基础设施建设全面推进，实施农业综合开发项目56个，修建基本农田35.42万亩，铺设广电线路1107公里。建设新农村示范村210个、扶贫重点村707个，实施“四改三建”11.08万户，修建“四田”36.08万亩，增加有效灌溉面积16.11万亩，发展节水灌溉17.24万亩，实施农村饮水工程1010处，解决了62.68万人的饮水安全问题。

城镇建设力度加大，城乡面貌明显改观。完成了中心城市总体规划修编，编制了一批专项规划和控制性、修建性详细规划，城市规划覆盖率达到65%，比2005年提高35个百分点。城市建设步伐加快，累计完成投资27.82亿元，其中中心城市投资14.76亿元，建成了丹江公园、江滨大道、市区南大门、商鞅广场、龟山风景区一期和丹江立交桥改造等市区重点工程，行政中心、体育中心、丹南新区基础设施、莲湖公园南扩和西商二线城区连接线等重点建设工程快速推进，倍受社会关注的环城北路开工建设，新建市区道路18条33公里，改造25条14公里，市区面积由18平方公里扩大到25平方公里。六县县城和重点镇建设扎实推进，2010年城镇化水平达到36%，五年提高了13.8个百分点。出台了鼓励农村居民进城落户的11个配套政策，农村居民进城落户工作有序开展。城乡联创成效明显，柞水县被命名为国家卫生县城，镇安、洛南两县被命名为省级卫生县城，商南县创建省级卫生县城通过验收，中心城市创建成为省级园林城市，创建省级卫生城市通过考核验收。

生态建设扎实推进，环境保护成效显著。以小流域综合治理、退耕还林、天然林保护、节能减排和重点区域整治为主要内容的生态环境建设深入推进，五年造林绿化181.37万亩，其中重点区域绿化30万亩，森林覆盖率由52.4%提高到62.3%。完成了二龙山、南秦等14座病险水库除险加固，累计实施小流域综合治理138条，治理水土流失3925.5平方公里。市医疗废物、生活垃圾和污水处理厂建成运营，6县污水处理厂全面开工，关闭“五小”企业17个，淘汰落后水泥产能160万吨，单位生产总值能耗降低20%左右，二氧化硫和化学需氧量排放同比削减1.1％和7.3％，均超额完成省上下达的任务。去年中心城区空气质量达到或好于二级的天数为350天，比2005年增加60天，绿色生态成为商洛最靓丽的名片。

改革开放不断深入，发展动力显著增强。部门预算、国库集中支付改革全面推进，国有资产管理体制改革成效明显。集体林权、土地流转、粮食流通、种子管理、基层供销社、水务体制和农技服务体系改革进展顺利。医药卫生体制改革取得重大进展，药品“三统一”覆盖所有基层医疗单位，镇安县综合医改试点工作扎实推进。文化体制改革步伐加快，市直文化单位改革基本到位。投融资平台建设取得实质进展，成立了市城投公司、中小企业担保公司和创投公司。市县区政府机构改革顺利完成，乡镇机构改革有序推进。对外开放和招商引资成效显著，成功引进比亚迪、中国黄金、陕西延长、国电集团、华电集团、陕煤化集团、肯德基、华润万家等知名大企业落户商洛，马来西亚客商在商洛设立了全省首家外商独资投资性公司，五年签约招商项目740个，引资到位资金239.96亿元，是“十五”的8.9倍。实际利用外资1.3亿美元，实现进出口贸易4.5亿美元，较“十五”分别增长25倍和35倍。非公有制经济不断壮大，去年占GDP的比重达到48.6%，较2005年提高4.4个百分点。

社会事业全面进步，民生民本持续改善。义务教育实现城乡免费，职业教育规模扩大，民办教育健康发展，高等教育质量不断提高，高考二本以上上线率连续三年居全省第一，“蛋奶工程”惠及18.4万寄宿制中小学生和农村幼儿。科技创新步伐加快，累计获得省政府科技奖8项、省级专利奖2项。建设标准化村卫生室2232个，新农合参合率达到95.9%，连续七年居全省第一。人口和计划生育政策全面落实，“十一五”末人口自然增长率控制在 2.85‰以内。文化事业快速发展，广播、电视覆盖率分别达到93.6%和98.7%。实施体育惠民建设项目316个，全民健身服务体系日趋完善。集中财力实施民生八大工程，累计建设民生项目11563个、投资103.6亿元，加快了公共服务均等化进程。城乡居民住房条件有了较大改善，建设经济适用房80.39万平方米、廉租房14.45万平方米，发放租赁补贴9171户，实施农村旧房改造11478户，解决了11033户低收入群众的住房困难。城镇居民养老保险和基本医疗保险从无到有，新型农村社会养老保险试点深入推进，城乡居民最低生活保障应保尽保，高龄老人生活补贴制度开始实施，职工养老金、新农合补助、城市低保、农村低保、五保户供养标准分别提高167%、500%、107%、156%和344 %。制定出台了残疾人普惠特惠政策，1.91万农村特困残疾人享受到了政府定期定额生活补贴。就业形势基本稳定，全市新增就业岗位5.2万个，累计转移农村劳动力271.4万人次，城镇登记失业率控制在4%以内。实施工赈、扶贫和生态移民17511户74604人，41.25万低收入人口实现脱贫。

民主法治进程加快，政府管理全面加强。自觉接受人民代表大会及其常委会的法律监督和人民政协的民主监督，支持各民主党派、工商联、无党派人士和人民团体参政议政，五年办理人大代表建议698件、政协提案1111件。认真实施行政许可法和《全面推进依法行政实施纲要》，进一步规范了行政审批程序，废止规范性文件261件、修改154件。积极开展依法行政示范县和示范单位创建活动，“五五”普法顺利通过省上验收，依法治市工作深入推进。完成了第六次、七次村委会换届选举，“一册两聚四会”制度全面推行，村务公开和民主管理工作稳步推进。积极推行乡镇公务接待廉政灶制度，强化行政监察和审计监督，政府系统廉政建设成效显著。深入开展“五型机关”创建和“工作落实年”、“企业服务年”活动，各级干部作风明显改进，行政效能和服务水平显著提升。在全省率先建立健全了县区消防队站，组建了综合应急救援队伍，应急处置能力不断增强。“平安商洛”创建活动深入开展，人民群众对社会治安的满意率连续五年居全省第一，建成了县区信访联合接待大厅，群众反映的突出问题基本得到及时解决，全市安全生产形势总体平稳。

统计、工商、地震、气象、水文、民族、宗教、侨务、邮政、烟草、盐务、保险、老龄、方志、档案、人防、慈善、红十字会等工作都取得了新的成绩。

各位代表，过去的五年，我们经受住了众多挑战和考验，取得了令人瞩目的成就。这是省委、省政府和市委正确领导的结果，是市人大、市政协支持监督的结果，是全市人民同心同德、顽强拼搏的结果。在此，我代表市人民政府，向全市广大干部群众、驻商部队指战员、武警官兵和公安干警致以崇高的敬意！向大力支持政府工作的各位代表、各位委员和各界人士表示衷心的感谢！

在充分肯定成绩的同时，我们也清醒地看到发展中存在的矛盾和问题：一是我市经济仍然欠发达，总量偏小、发展不足的问题突出；二是资源约束和环境压力仍然较大，粗放型发展方式尚未根本扭转，转变发展方式需要付出艰辛努力；三是城乡居民特别是农民收入低，农业现代化、工业化和城镇化水平低，制约着经济社会快速发展；四是公共服务水平有待提高，改善民生的任务依然艰巨；五是政府工作中的官僚主义、形式主义和奢侈浪费现象不同程度存在，行政效率和服务水平有待进一步提高。对于这些问题，我们将高度重视，采取有效措施加以解决，绝不辜负全市人民的重托和期望。

二、“十二五”的总体思路和目标任务

“十二五”时期，是我市加快转变经济发展方式的攻坚时期，也是加快推进率先突破发展的关键时期。尽管面临诸多风险和挑战，但也面临着国家深入推进新一轮西部大开发、加快实施关天经济区发展规划、省上大力推动三大区域协调发展的重大历史机遇。更为重要的是，经过多年的努力，全市特色产业不断壮大，基础设施明显改善，区位优势日益凸现，发展环境越来越好。站在新的历史起点，面对新的机遇和挑战，我们必须增强责任感和紧迫感，坚定信心，下定决心，加快发展，循环发展，创新发展，协调发展，奋力开创商洛经济社会发展的新局面。

未来五年全市经济社会发展的总体思路是：高举中国特色社会主义伟大旗帜，以邓小平理论和“三个代表”重要思想为指导，深入贯彻落实科学发展观，进一步解放思想、转变观念，抓住难得的发展机遇，以科学发展和富民强市为主题，以发展循环经济为主线，以保障和改善民生为出发点和落脚点，继续坚持生态立市、产业兴市、工业强市，实施开放带动、城镇带动、项目带动、科技带动，切实转变发展方式，不断优化经济结构，着力提高发展质量，全力建设“三区四基地”，奋力推进率先突破发展，为全面建设小康社会奠定坚实基础。

未来五年的主要目标是：重点工作实现“三个上水平”，主要指标实现“四个翻番”，经济社会初步实现率先突破发展。“三个上水平”：即循环经济发展、民生和公共服务保障、区域创新和开放三项工作上水平。“四个翻番”：即生产总值、财政收入、城乡居民收入、固定资产投资分别翻一番，生产总值达到650亿元，是2010年的2.37倍；财政总收入达到50亿元，是2010年的2.35倍；城乡居民收入分别达到30000元和8000元，分别是2010年的2.03倍和2.22倍，赶上全省平均水平；全社会固定资产投资累计达到2000亿元，是“十一五”的2.3倍以上。

未来五年的主要任务是：

(一)着力构建现代优势特色产业体系。按照存量调结构腾空间、增量优结构扩空间的原则，处理好现有经济结构和新增产业项目的关系，进一步促进工业化与信息化深度融合，加快培育新材料、先进装备制造、节能环保等战略性新兴产业，大力发展劳务输出、特色文化、现代物流、金融保险等服务业，做大做强现代材料、生态旅游、生物医药、绿色食品等优势特色产业，增强现代农业对农民增收的引领作用。到“十二五”末，建成以比亚迪太阳能光伏为龙头的1个200亿元产值产业，以铁、钒、钼系列开发为主的3个100亿元产值产业，以锌、镁、石墨、电力、绿色食品系列开发为主的5个50亿元产值产业，以黄金、环保、氟材料、钾长石、生物医药、生态旅游、汽车零部件、服务业为主的8个20亿元以上产值产业，形成千亿级产业集群。全市规模以上企业发展到220户，全部工业增加值达到260亿元，比2010年翻两番，对地方财政的贡献率达到60％以上；服务业增加值超过260亿元，占GDP的比重达到40%。

（二）着力创建全国循环经济示范城市。坚持循环、绿色、低碳的发展理念，把加快循环经济发展贯穿经济社会发展的全过程和各个领域，逐步形成企业循环式发展、产业循环式融合、资源循环式利用、区域循环式开发的循环经济体系，走出一条产业集聚、企业集中、资源集约和低耗、减排、高效的发展路子。重点建设10大循环经济产业链，实施20个循环经济标志性工程，加快培育1个国家级10个省级50个市级循环经济示范企业，着力建成3个省级5个市级循环经济园区，把商洛建成全国循环经济示范区和全国尾矿综合利用示范基地。抓好绿色村庄、绿色城镇、绿色河流等绿色生态工程建设，积极实施农村废弃物综合利用工程，梯次推进循环型社会建设。

（三）着力打造关天经济区次核心城市。坚持“做美城市、做强县城、做大集镇、做好社区”的方略，形成以中心城市为核心、六县县城为重点、重点镇和建制镇为支撑的城镇体系。以建设山水园林生态旅游城市和西安第二生活区为目标，按照“一轴两山三城”发展格局，全力抓好中心城市建设，扎实推进绕城环线、商鞅大道、丹南片区、南秦片区建设，大力实施旧城改造和“城中村”改造，加快城市公园及“五化”工程建设，进一步拉大城市框架，完善城市功能，丰富城市内涵，提升城市品位。到2015年末，中心城市面积超过40平方公里，人口超过40万人。加快商丹一体化建设步伐，建成商丹城市一级主干道，加强丹江流域综合整治，形成百里绿色景观带；加快亚迪城、商棣工业园、丹凤东河产业园建设，形成相互配套的产业支撑体系；加快沙河子、棣花等重点镇建设，形成商丹谷地串珠式城镇群。全面落实鼓励农村居民进城落户的一系列政策，力争“十二五”末，有40万农村居民到城镇安居乐业，全市城镇化水平达到48%。

（四）着力加强基础设施和生态建设。以高速公路、铁路复线、高速铁路、国省道改造、产业路和农村公路建设为重点，加快构建现代化综合交通网络。到“十二五”末，实现县县通高速，乡乡通油路，100%行政村和60%自然村通水泥路，100%乡镇和90%以上行政村通班车。积极做好西安商洛机场项目前期工作，力争开工建设。加快商洛发电厂、热电厂等现代材料基地配套能源工程建设，完善330千伏及以下电网布局，加快农村电气化建设，提高供电保障能力。加快中小河流治理、病险水库除险加固和重点水源工程建设，进一步强化防灾减灾综合体系建设和水资源供需保障。加快实施“三网”融合，推进信息化建设进程。合理利用和保护资源，加大节能减排力度，搞好重点地区生态保护，把商洛建成关天经济区生态发展示范区。

（五）着力构建创新开放型区域。全面落实中长期科技、教育、人才规划纲要，大力实施知识产权战略，实施科技资源统筹创新工程，加快建立完善以企业为主体、市场为导向、大专院校和科研院所为支撑、产学研一体化的技术创新体系，提升科技创新能力，增强科技支撑引领作用。积极营造各种所有制经济依法平等使用生产要素、公平参与市场竞争、同等受到法律保护的制度环境，“十二五”末非公有制经济比重达到55%左右。深化行政管理体制和社会领域各项改革，坚持“引进来”与“走出去”相结合，进一步拓展对外合作范围，大幅提高经济外向度。到“十二五”末，累计实现招商引资到位资金900亿元，进出口贸易16亿美元，年均增长15%以上。

（六）着力促进基本公共服务均等化。持续加大民生工程建设投入，今后五年财政增收部分主要用于民生，大力实施促进就业、收入倍增、全民社保、教育提升、卫生健康、文化惠民、保障住房、消除贫困、改善环境、服务生产等十大领域民生工程，努力做到公共服务水平与经济发展相适应、与群众不断增长的需求相适应，让商洛人民生活得更加幸福、更有尊严。基本养老两年实现城乡全覆盖，并形成保障标准逐年提高的机制。加大保障性住房、棚户区改造和农村危房改造力度，五年解决9万余户城乡中低收入居民的住房困难。优先发展教育事业，逐步增加财政对教育的投入，稳步提升全民受教育程度。深化医疗卫生体制改革，加快医疗卫生服务体系建设，实现小病不出乡、大病不出县、预防在基层、公共卫生服务城乡一体化。实施深山危居整体搬迁工程，用十年时间搬迁人口71万人。加强社会管理能力建设，持续推进平安创建工作，切实维护社会和谐稳定。

三、2011年经济社会发展的重点工作

今年是实施“十二五”规划的开局之年，也是本届政府的收官之年。做好今年的各项工作，继续保持又好又快发展态势，对于实现“十二五”高点起步、良好开局具有十分重要的意义。全市经济社会发展的主要预期目标是：生产总值增长13.5%左右，财政总收入增长18%，地方财政收入增长18%，城镇居民人均可支配收入和农民人均纯收入分别增长15%和16%左右，城镇登记失业率控制在4.5%以内，居民消费价格涨幅控制在4%左右，人口自然增长率控制在5.5‰以内，节能减排指标控制在省上规定范围内。

（一）持续实施项目带动，保持投资快速增长

加快推进重点项目建设。全年安排市级重点项目150个，年度投资200亿元以上，其中产业类项目79个，重大基础设施项目15个，确保全社会固定资产投资增长25%。全市筹措项目前期费1亿元，其中每县区不低于1000万元，商丹园区不低于1500万元，策划重大前期项目300个，完成山阳生物质能源综合开发、镇安抽水蓄能电站和商洛热电厂等150个前期项目。建立健全重大项目与金融机构对接机制，鼓励通过BT、BOT和发行企业债券等方式扩大融资规模，努力解决制约项目建设的资金难题。认真落实《商洛市重大项目建设考评办法》，严格项目建设“六制”管理，全力加快项目建设进度。

进一步优化投资结构。充分发挥政府引导作用，建立国有、民间、外商等多元化的投资稳定增长机制。综合运用产业、土地、金融、节能、环保等政策，加大民生投入力度，提高产业投资比重，重点向节能环保、新材料、高新技术和战略性新兴产业倾斜。严格控制高耗能、高污染和产能过剩的行业投资，防止各种类型的重复建设。继续扩大各级财政资金投入，切实加强各类专项资金整合，增强政府投资的导向作用和乘数效应。

切实加强基础设施建设。扎实做好西合、西康铁路增建二线，以及西商二线、洛商高速建设的环境保障工作，力争山柞高速全线开工建设。抓好203省道山阳宽坪至漫川关、102省道柞水溶洞段和镇安过境公路等干线公路改建工程，建成通村水泥路100公里、通乡油路200公里。完成市区和金丝峡两个一级汽车客运站年度建设任务，建成镇安和柞水两个二级客运站、山阳漫川三级站和7个乡镇客运站。启动建设330千伏张村变增容扩建工程，建成投运110千伏张村变至比亚迪供电及西康铁路复线供电工程，加快35千伏罗庄变升压改造工程进度。推进农村电网升级改造，建设改造10千伏线路412公里、0.4千伏线路679公里，行政村网改面由86%提高到92%。

（二）坚持率先发展工业，加快新型工业化进程

做大工业总量。抓好延长氟化工一期、五洲公司钒系列开发等48个工业项目建设，年内完成投资73.5亿元，其中九龙万吨钼采选、延长甲醇汽柴油等15个项目建成投产，当年新增产能70亿元。落实“一企一策”包抓措施，加大对炼锌厂、大西沟铁矿、千家坪钒矿、盘龙制药等骨干企业的扶持力度，确保全市规模以上工业增加值增长30%左右。鼓励企业重组和产权多元化，通过资本市场直接融资，对成功上市的企业给予重奖。加快国有企业股份制改造步伐，积极稳妥地推进氮肥厂、运输公司和造纸厂等国有企业改革重组，转换经营机制，盘活存量资产。

调整工业结构。优化要素资源配置，推广循环发展模式，加快构建结构优化、技术先进、清洁安全、附加值高的现代产业体系。围绕发展壮大有色金属、钢铁、化工材料等优势特色产业，扎实推进矿产资源整合，促进矿产资源集约化、规模化利用。运用现代信息技术和先进实用技术，加快改造矿产金属、建材化工、绿色食品、生物医药等传统产业，推进资源深度转化，增强市场竞争力。积极培育战略性新兴产业，支持发展新材料、生物技术、信息技术、节能环保等产业，重点在太阳能光伏产业方面下工夫，建成以比亚迪、延长氟化工为核心的生产基地，形成销售收入过百亿元的产能。

强化产业集聚。坚持合理布局、注重特色的原则，统筹安排商丹园区和县域工业集中区项目，形成园区之间相互支撑、相互配套的完整产业体系，使园区真正成为工业发展的主引擎。加快商丹园区建设步伐，扎实推进8个基础设施和15个重点产业项目建设，启动建设工业园区铁路专线和煤炭转运站，比亚迪1000兆瓦太阳能电池、延长氟化工一期、陕西盘龙医药保健品加工、福建众森可生物降解塑料一期等项目建成投产。坚持“一把手”主抓机制，全面推进县域工业集中区规划、机构、基础设施、产业发展和配套体系建设，创新园区管理体制机制，全年新增入园企业40户。

推进自主创新。全面启动商洛国家现代材料特色产业基地和科技特色产业示范基地申报工作。完成陕西材料工程研究院商洛分院、陕西循环经济工程研究院商洛分院、中国尾矿资源利用中心商洛中心建设任务，继续抓好中国·商洛核桃研究所建设，引导企业与高校、院所搭建各种形式的创新平台。从今年起市级科技经费主要用于支持企业技术创新。鼓励企业加快新产品开发和技术改造，全市培育新科技成果20项、专利申请100件。加强“科技新星”青年科技人才培养，实行中小企业首席工程师制度，全面推行科技特派员基层创业行动。

（三）大力发展现代农业，提升农业产业化水平

积极发展农业优势产业。按照“扩经、强牧、优果、兴药”的思路，持续推进“一村一品”、“一乡一业”，重点扶持中药材、林果、畜牧和设施蔬菜等四大优势产业，加快建设一批富有商洛特色的现代农业产业园。加强商洛道地药材药源基地建设，新增中药材种植10万亩。加快核桃良种基地县建设，大力发展以核桃、板栗为主的林果产业，全年新增核桃、板栗基地30万亩，良种嫁接改造30万亩，促进林果产业由数量增长型向质量效益型转变。抓好百万头生猪大县、10个十万头生猪乡镇和20个万头生猪示范村建设，年出栏生猪130万头、家禽500万只。下大气力抓好“菜篮子”工程，积极发展反季节菜与大路菜相结合的蔬菜产业，因地制宜建设县城和乡镇蔬菜自给供应基地，抓好商州万亩设施蔬菜基地建设，全年新发展设施蔬菜7000亩。加快发展区域特色产业，新增茶园3万亩，种植烤烟10万亩。积极推广高效增产技术，提高粮食单产，确保粮食总产55万吨左右。继续实施“人人技能工程”，大力发展劳务经济，组织劳务输出50万人，创收30亿元。

加快农业产业化进程。积极推进与台湾中卫发展中心的合作，加快建设秦岭有机生物园，促进以丹参为主的中药产业深度开发。继续加强与国内知名龙头企业的联系，积极引进战略合作伙伴，借助其技术和品牌优势，推动农业优势产业和现有企业整合。加大龙头企业培育力度，申报认定省级龙头企业5家以上，启动国家级龙头企业申报工作。加强农产品质量安全监管体系建设，加大“三品”申报认证力度，全年认定10个基地，认证10个产品，注册7个农产品商标。推行“企业+协会+农户”的产业化模式，抓好50个市级专业合作示范社建设，全面提升农业产业化经营水平。

改善农业农村基本条件。把农田水利作为农村基础设施建设的重点任务，把落实最严格的水资源管理制度作为转变经济发展方式的战略举措，突出加强农田水利等薄弱环节建设，力争今年水利投入比上年增加一倍以上。以提高农业灌溉能力为目标，大力实施农田水利工程，建设基本农田9万亩，新修“四田”4万亩，改造提高3万亩，新增灌溉面积2万亩，发展节水灌溉3万亩。以提高城乡供水安全保障能力为目标，加快重点水源工程建设，搞好商州庙湾和洛南姬家河水库的除险加固。以提高防灾减灾能力为目标，加强气象服务体系和水文基础设施建设，加快丹江口库区及上游水土保持工程建设步伐，加强山阳马滩河、商州南秦河等8条中小河流治理，治理水土流失1000平方公里。扎实抓好72个新农村试点示范村建设。加强地质灾害防治工作，治理地质灾害隐患点16处。按照小城镇建设、现代农业发展和扶贫搬迁三位一体的思路，搞好移民搬迁工作，建设移民新村30个，搬迁人口1.5万人。实施扶贫开发连片项目区10个，建设扶贫重点村50个，使6万贫困人口稳定脱贫。扎实推进灾后重建工作，5月底前完成55处灾民集中安置点建设任务。

（四）大力发展第三产业，促进消费快速增长

做大做强生态旅游业。按照“政府主导、企业主体、有序开发、错位发展”的思路，加快旅游产业发展步伐，促进全市旅游转型升级。加大重点旅游景区建设力度，快速推进仙娥湖生态旅游度假区开发，天竺山、塔云山、九天山等景区“五·一”前开园。加快完善旅游基础设施，积极发展旅行社、星级酒店、旅游纪念品、旅游购物、旅游咨询服务和文化休闲娱乐等旅游配套产业，全面提升旅游综合服务水平。大力培育演艺团体和企业，推进文化演艺进景区。加强旅游行业管理，维护旅游市场秩序。抓好省级旅游示范村建设，规范发展乡村旅游。以打造“秦岭最美是商洛”品牌为重点，精心筹办第二届中国秦岭生态旅游节，加强旅游宣传促销，积极开拓客源市场，全年接待游客1500万人次，实现综合收入60亿元。

加快促进服务业发展。运用现代服务技术和经营方式，改造提升商贸、餐饮等传统服务业，启动天成万豪酒店建设，商洛晨光城、通江国际电子数码城和市区桂园、东关、州城路3个综合市场年内建成运营。大力发展信息、物流、金融、研发设计、服务外包等生产性服务业，培育发展会展、保险、文化传媒等现代服务业。积极发展电子商务、连锁经营和物流配送，加快商山物流园、镇安百利得超市物流中心建设，培育一批具有先进经营理念的现代物流企业。改善金融生态环境，加快组建小额信贷公司，切实发挥担保公司作用，全年新增贷款30亿元，力争服务业增加值增长15%。

进一步扩大消费需求。采取超常规措施推进城乡居民增加收入，从今年起逐步提高财政支出用于促进消费的比重，重点用于增加城镇低收入群体和农民的补贴。全面落实各项惠农补贴政策，深入实施农民增收工程，大力推进农村土地经营权流转，千方百计增加农民收入。健全职工工资正常增长和支付保障机制，完善事业单位收入分配制度，增加农村优抚对象和生活困难群众补助，着力解决好农民工工资拖欠问题，做到城乡居民收入增长与经济发展同步，进一步增强消费能力，扩大城乡消费预期。积极开发休闲、娱乐、体育、保健等新型消费业态，稳定发展住房、汽车等大宗消费，扩大网上购物和信用消费，推动消费结构优化升级。深入实施“万村千乡市场工程”，健全城乡商贸流通体系，新建和改造5个县级农贸市场。继续做好家电、汽车、摩托车、农机具下乡工作，全年力争通过家电下乡多销家电5000台。提高价格监测预警水平，加强市场监督管理，完善市场营销体系，营造良好消费环境，确保社会消费品零售总额增长19%。

（五）加快城镇化进程，统筹城乡协调发展

发挥规划引领控制作用。坚持城镇总体规划、产业发展规划和土地利用规划“三规合一”原则，加大规划资金投入力度，科学编制《商洛市“十二五”城镇化规划》、商丹一体化控制性详细规划、中心城市四大门户区城市设计及城市雕塑规划，新城区发展和老城区改造控制性详细规划，力争中心城区详细规划覆盖率达到80%以上。运用规划把脉活动成果，修编《商洛市城镇体系规划》，全面完成城乡一体化规划，超前编制乡镇基础设施建设等专项规划，编制完成重点镇和70个示范村建设规划，增强各层次、各领域规划的协调性和可实施性，力争县城详细规划覆盖率达到85%。健全完善规划管理体制机制，加大规划实施和监管力度，严肃查处各种违法建设行为。

加快中心城市建设步伐。坚持以市场化推进城镇化建设，通过资本金注入、优质资产划入、特许经营权授让、一级土地市场开发等方式，壮大城市建设投融资平台实力。加快城市重点项目建设，启动北新街东延、商鞅大道、市文化艺术中心和城中村改造等项目建设，完成环城北路、惠民小区二期主体工程，西商二线城区连接线工程建成通车，西街片区旧城改造和莲湖公园东侧改造项目完成安置房主体工程，工农路南段改造项目启动征地拆迁工作，龟山风景区二期、金凤山民俗风情园完成绿化、美化和亮化，市体育馆“五·一”开馆，莲湖公园南扩“十·一”开园。加快城区“退二进三”和“优化升级”步伐。巩固扩大中心城市创卫成果，加快完善环卫基础设施，启动建设城市信息化管理工程，全面提升城市精细化管理水平。

加强县城和重点镇建设。按照政府引领、市场融资、项目带动的原则，以建设大县城、推进城镇化为目标，从扩大城镇规模、强化宜居功能、提升管理水平三个层次入手，实施村镇整合、以城带镇发展、产业园区建设、城镇社区建设、环境综合治理、强化管理服务六大工程，支持六县加快县城建设，全面改善基础设施条件，增强产业发展和人口聚集功能，促进县域经济快速发展。全力推进重点镇建设，力争两个镇列入30个省级重点示范镇，深化重点镇综合改革，完善重点镇投融资体制，加快镇区基础设施建设步伐。

全力推进城乡统筹发展。全面推进商丹一体化建设，启动312国道商州至丹凤一级公路改建工程，实施丹江流域综合治理，加快完善沿线金融、商贸等配套设施。深化户籍制度改革，坚持自愿原则，积极引导农村居民进城落户，着力解决进城落户居民的住房、就业、就医和子女上学等现实问题。加快推进商州—丹凤城乡统筹示范区建设，力争全市城镇化水平达到39%。积极稳妥推进撤乡并镇工作，鼓励和支持基础薄弱、人口持续减少的村合并，优化居民点布局，发展新型农村社区，建设农民幸福家园。依法搞好第八次村委会换届选举工作。加强少数民族地区工作，扶持少数民族地区建设和发展。深入推进“城乡联创”，争取丹凤通过省级卫生县城验收，洛南通过省级卫生县城第二次复审，推进柞水省级生态县、镇安省级园林县城和国家级卫生县城创建，启动商南、镇安省级生态县创建工作。

（六）加强资源节约和环境保护，提高生态文明水平

加快推进循环经济发展。认真实施《陕南循环经济产业发展规划商洛市实施意见》，加强规划指导，强化政策支持，发展新型环保产业，增强可持续发展能力。继续抓好省市循环经济试点单位建设，做好商丹循环工业经济园区和商洛炼锌厂申报省级循环经济示范单位、尧柏秀山水泥公司申报国家级循环经济示范企业认定工作。启动钼材料及钼金属制品循环发展产业链、煤电及钒矿低热煤发电循环发展产业链建设，加快16个在建循环经济标志性工程建设进度，全力支持清洁生产示范企业建设。抓好镇安水源预制公司铅锌尾矿加气混凝土砌块等10个尾矿综合利用项目。大力推行秸秆综合利用，扩大联户沼气和养殖小区沼气规模，推广“果—畜—沼—菜”四位一体生产模式，形成产业纵向延伸、横向拓展，资源、产业和产品多层面联动的发展格局。

加强环境保护和生态建设。按照“控制总量、削减存量、消化增量”的要求，强力推进节能减排，加快淘汰落后产能、工艺装置和技术设备，切实加大对污染减排的管理，严格控制水泥行业和机动车氮氧化合物排放。完成六县污水和垃圾处理设施建设任务，提高污水、垃圾处理率。继续实施天然林保护、退耕还林等工程，深入开展全民植树造林活动，新增工程造林30万亩，其中飞播造林12万亩。启动中心城市创建省级环保模范城市工作，加快中心城区、六县县城周边以及公路、铁路沿线绿化步伐，完成重点区域绿化2.5万亩。全面启动农村清洁工程，大力实施农村环境综合整治，进一步改善环境质量，确保中心城区空气质量达到或好于二级的天数保持在350天以上。

合理利用和节约资源。加强矿产资源地质勘查，完善资源开发和生态环境补偿机制。综合运用工程节能、技术节能和合同能源管理三大措施，重点抓好20家耗能大户的节能降耗，实施6个工业企业重大节能技术改造示范项目，积极推广节能新技术、新产品，鼓励发展水电、太阳能等绿色能源，逐步降低煤炭消费比例。坚持总量控制和定额管理相结合，加强水资源配置管理，节约生产生活用水。严格执行土地利用总体规划，认真落实建设用地增减挂钩、耕地占补平衡政策，扎实开展土地卫片执法和专项检查，加强土地复垦和空心村治理，推广紧凑节地的发展模式，实现土地节约集约利用。

（七）进一步深化改革开放，增强经济发展活力

稳步推进各项改革。深化行政管理体制改革，完成乡镇机构改革任务。完善国有资产管理体制，健全国有资本经营预算和收益分享制度。加快医药卫生体制改革步伐，规范完善药品配送机制，合理调整药品“三统一”补偿标准并确保及时足额到位，继续推进镇安县综合医改和公立医院改革试点工作。以健全聘用制度和岗位管理制度为重点，分类推进事业单位改革。积极推进投融资体制改革，抓好土地经营权、林权、采矿权质押贷款试点，推动金融产品、抵押担保方式和金融服务方式创新。继续改革财政资金投入方式，产业项目逐步采取资本金注入、贴息、担保等方式投入，事业性投入要打破部门分割、适当整合，提高使用效益。加快集体林权制度配套改革，实现林业规模化产业化发展。

深度开展招商引资。全方位推进对外开放，善借“外脑”，广用“外力”，促进发展。加强投资环境建设，切实保障投资者和经营者的合法权益。继续加大招商引资力度，大力开展以商招商、以企招商和产业链招商，综合考量项目的投资强度、销售收入和税收贡献率，最大限度提高项目引进效益。积极组团参加西洽会、农高会等招商活动，定期赴长三角、珠三角等地叩门招商，努力引进一批重大产业项目和大型企业。继续深化“企业服务年”活动，建立客商和企业落户服务机制，促使招商项目顺利实施，力争全年招商引资到位资金130亿元，其中商丹园区50亿元。

扩大对外经济贸易。大力实施“走出去”战略，加快发展外向型经济，力争全年外贸进出口总额达到2.8亿美元，增长15％。积极应对外贸出口新形势，用足用好外贸扶持政策，重点扶持商洛比亚迪、炼锌厂、西部鑫兴钼业等骨干出口企业，新增外贸出口企业3-5家。加快转变外贸发展方式，调整优化出口产品结构，巩固太阳能电池、锌锭、钼粉等特色产品的出口优势，扩大有色金属产品和高新技术产品出口份额，扶持农产品加工企业取得自营出口权。

加快发展非公经济。以中小企业为重点，培育壮大各类市场主体，促进非公有制经济快速发展。认真落实《国务院关于鼓励和引导民间投资健康发展的若干意见》，结合商洛实际制定出台实施意见。坚持非禁即入、降低门槛，鼓励民间资本进入基础产业、基础设施、金融服务和商贸流通领域，引导民间资本参与政策性住房、社会事业和市政公共事业等发展，让非公有制企业平等享受政府公共资源。大力倡导全民创业，支持家庭创业和自主创业，全年新增私营企业300户、个体工商户2500户。

（八）大力发展社会事业，全力保障和改善民生

加快发展社会事业。积极发展学前教育，加快中心幼儿园建设。继续实施中小学校舍安全工程，搞好学校布局调整和标准化建设，完成丹凤县义务教育均衡发展合格县创建任务。优化高中教育资源配置，提升高中教育办学水平。加快职业教育资源整合，推进商洛职业技术学院快速发展。支持高等教育健康发展，启动省市共建商洛学院。积极发展继续教育，大力支持民办教育。强化师资队伍建设，加强教育改革创新，持续提高教育质量。加快城乡医疗卫生服务体系建设，重点抓好4所县级医院、11个乡镇中心卫生院、2个城市社区卫生服务中心标准化建设。加强突发公共卫生事件处置能力建设，实施好重大传染病和地方病、慢性非传染性疾病防治等公共卫生项目，组织好65岁以上老人健康检查。继续深化文化体制改革，推进文化事业和文化产业发展。进一步完善广播电视“村村通”、文化资源共享、乡镇文化站、村（社区）文化活动室和农家书屋等公共文化设施，加快城镇有线电视数字化整体转换步伐。搞好第六次人口普查，继续稳定适度的低生育水平，综合治理出生人口性别比偏高问题，大力实施母亲健康和优生促进工程，提高出生人口素质，促进人口均衡发展。加强公共体育设施建设，启动市游泳馆和体育场建设工程，改造商南县体育场，建成丹凤、山阳两县体育中心。广泛开展群众性体育活动，支持发展有特色、能形成优势的竞技体育项目，提升全市竞技体育整体水平。

深入实施民生工程。全市安排民生项目89类、投资38亿元，其中基本建设类项目投资22.5亿元。继续为群众办好十件实事：一是加快保障性住房建设，完成投资12亿元，建设廉租房6000套、公共租赁房8000套、经济适用房3000套、限价商品房6000套，改造棚户区9000套。二是抓好农村安全饮水工作，实施安全饮水工程200处，解决16万人的饮水安全问题。三是全面加强就业工作，建立和完善促进就业的综合政策支持体系，重点做好高校毕业生、进城落户农村居民、农村退伍军人和城镇失业人员的就业工作，新增城镇就业1.2万人，下岗失业人员再就业5000人，农村劳动力转移就业58.2万人。四是大力推进社保“扩面提标”，基本养老保险扩面新增4000人，企业离退休人员基本养老金水平提高10%。五是扩大残疾人特惠政策覆盖面，加强残疾人服务体系建设，使3.5万名残疾人直接受益。六是启动市幼儿园改扩建工程，着力解决市区入园难问题。七是完善基本医疗保险政策，城镇职工和城镇居民医保住院医疗费政策范围内报销比例分别提高到70%和50%以上，新农合住院报销比例提高到70%。八是加强农村五保供养工作，完成3个县级中心敬老院和4个区域敬老院建设，全市五保集中供养率提高到50%。九是建立高龄老人补贴制度，为全市3.1万老年人提供生活补贴。十是提高新农合和城镇居民基本医疗保险财政补助标准，今年人均分别提高80元。

完善社会保障体系。加快推进覆盖城乡居民的社会保障体系建设，扩大各项社会保障制度覆盖面，做到各类群体应保尽保。完善城乡低保、五保供养制度，规范临时救助制度，加强城乡医疗救助，实行孤儿发放基本生活费制度，提高孤老孤残孤儿等特殊群体的保障水平。鼓励商业保险公司提供与社会保险相衔接的产品与服务，促进各保险公司不断扩大保险范围，为现代农业发展提供有效保障。大力推进农村政策性房屋保险和农村小额人身保险，政策性农村房屋保险参保率达到80%。统筹财政、企业和社会资金，用于廉租房和公共租赁住房建设、棚户区改造、农村危房改造补助，支持各县区和乡镇建设经济适用房，重点解决乡镇公益性服务人员和农村进城落户居民的住房问题。做好老龄、青少年和妇女儿童权益保障工作，加快发展社会福利、红十字会和慈善事业。

（九）深入推进平安创建，促进社会和谐稳定

全面加强维稳工作。编制完成应急建设规划，提升危机预警水平，启动公安消防特警队站建设，完善救援队伍和装备，加大应急物资储备，推进基层应急管理，提高社会公众防灾避险和互救能力。加大基层派出所、司法所建设力度，加强社会治安综合治理，依法打击各类违法犯罪，进一步提高群众的安全感。深入开展“六五”普法教育。深化信访“三无”县区和乡镇创建活动，健全规范信访联合接待工作机制，充分发挥人民调解组织作用，以解决群众实际问题为重点，妥善处理人民内部矛盾，全力维护社会稳定。

扎实抓好安全生产。加强安全生产体制机制建设，建立完善全市安全隐患数据库，落实政府监管责任和企业主体责任，继续深化道路交通、非煤矿山、烟花爆竹、危险化学品等重点行业领域的安全专项整治，坚决遏制重特大事故发生。强化食品药品安全监管，严查制售假冒伪劣商品行为。

支持驻商部队正规化建设。长期以来，驻商部队和武警官兵对商洛的改革发展给予了极大支持。在推进率先突破发展进程中，我们要一如既往地支持驻商部队的革命化、现代化、正规化建设，广泛开展双拥共建活动，加强民兵预备役基础设施建设，深入进行国防教育，进一步巩固和发展军政民团结的大好局面。

四、进一步加强政府自身建设

复杂多变的经济形势，繁重艰巨的发展任务，对政府工作提出了新的更高要求。我们要以深入开展“为民服务、创先争优”主题实践活动为契机，积极推进管理创新和服务创新，努力建设负责任、有作为、高效能、遵法纪、重廉洁的人民政府。

继续解放思想，激发政府工作的创造性。坚持把富民强市作为为政之首，紧跟时代脉搏和发展步伐，用开放理念、前瞻眼光和科学思维，创造性地开展工作，以新理念研究新问题，以新思路落实新任务，以新举措开创新局面。把转变职能和创新管理有机结合起来，积极推进政企分开、政事分开、事企分开、政府与市场中介组织分开，综合运用规划、产业政策和财税、价格等手段，努力提高政府经济调节和市场监管能力，强化社会管理和公共服务职能，统筹协调社会利益关系，着力增强发展的包容性和协调性。

坚持以人为本，增强亲民为民的主动性。坚持把改善民生作为为政之本，从群众最关心的事情抓起，从群众最困难的事情帮起，尽心竭力为群众办好事、办实事，做到政府工作始终围绕群众需求转，不断顺应人民群众的新期待。加快市县政务服务中心和乡镇便民服务中心建设，创新联系群众的方式方法，对基层和群众提出的问题，变上级督促处理为提前主动解决，在惠民服务中赢得民心，提高政府的公信力。

推进依法行政，确保施政行为的规范性。坚持把依法行政作为为政之纲，依法接受人大及其常委会的监督，积极支持人民政协履行职能，主动听取各民主党派、工商联和人民团体的意见和建议，认真办理人大代表议案、建议和政协委员提案。完善信息和新闻发布制度，主动接受新闻舆论监督和社会公众监督。健全行政执法体制，规范行政执法行为。深化行政审批制度改革，加快电子政务建设，深入推进政务公开，努力打造阳光政务。

改进工作作风，提高执行落实的时效性。坚持把强化效能作为为政之基，大力倡导“面对竞争有激情、面对困难有豪情、面对事业有热情”的工作精神，切实改进作风，提高行政效能，用实心做实事，用实干求实效。牢固树立“执行没有借口”的工作理念，强化政府执行力，实行任务分工负责，实干巧干、一线推进。各级领导干部带头提效率，改进文风会风，开短会、讲短话、发短文，尽量减少事务性应酬，腾出时间搞调研、抓协调、破难题。建立健全“凭结果说话”的考评体系，加大政府抓落实督查考核力度，严格实行过程督查制、结果通报制和责任追究制，确保政府工作有序推进、高效运转。

加强廉政建设，强化拒腐防变的自觉性。坚持把廉政勤政作为为政之要，认真贯彻执行党风廉政建设责任制和《廉政准则》，整体推进惩治和预防腐败体系建设各项工作。继续开展土地管理、工程建设、资源开发、产权转让、政府采购等领域的专项治理，扎实做好效能监察和审计监督，严肃查处各类违法违纪行为。坚持勤俭节约，反对铺张浪费，今年公务接待、公务用车和出国出境经费做到零增长。全体政府工作人员把自律作为基本要求，把严管作为最大爱护，清白做人、干净做事，树正气、做表率，聚民心、促发展。

各位代表，蓝图已经绘就，机遇催人奋进，使命就是责任。让我们高举中国特色社会主义伟大旗帜，深入贯彻落实科学发展观，在市委的坚强领导下，团结依靠全市人民，以百折不挠的意志、心无旁骛的信念、创新务实的作风，努力实现“十二五”的良好开端，以优异成绩迎接中国共产党成立90周年！
